# Insertion of N-Terminal Hinge Glycosylation Enhances Interactions of the Fc Region of Human IgG1 Monomers with Glycan-Dependent Receptors and Blocks Hemagglutination by the Influenza Virus

**DOI:** 10.4049/jimmunol.1801337

**Published:** 2019-01-25

**Authors:** Patricia A. Blundell, Dongli Lu, Mark Wilkinson, Anne Dell, Stuart Haslam, Richard J. Pleass

**Affiliations:** * Department of Parasitology, Liverpool School of Tropical Medicine, Liverpool L3 5QA, United Kingdom; and; † Department of Life Sciences, Imperial College London, London SW7 2AZ, United Kingdom

## Abstract

In therapeutic applications in which the Fc of IgG is critically important, the receptor binding and functional properties of the Fc are lost after deglycosylation or removal of the unique Asn^297^ N-X-(T/S) sequon. A population of Fcs bearing sialylated glycans has been identified as contributing to this functionality, and high levels of sialylation also lead to longer serum retention times advantageous for therapy. The efficacy of sialylated Fc has generated an incentive to modify the unique N-linked glycosylation site at Asn^297^, either through chemical and enzymatic methods or by mutagenesis of the Fc, that disrupts the protein–Asn^297^ carbohydrate interface. In this study, we took an alternative approach by inserting or deleting N-linked attachment sites into the body of the Fc to generate a portfolio of mutants with tailored effector functions. For example, we describe mutants with enhanced binding to low-affinity inhibitory human Fcγ and glycan receptors that may be usefully incorporated into existing Ab engineering approaches to treat or vaccinate against disease. The IgG1 Fc fragments containing complex sialylated glycans attached to the N-terminal Asn^221^ sequon bound influenza virus hemagglutinin and disrupted influenza A–mediated agglutination of human erythrocytes.

## Introduction

Multiple lines of evidence have shown that glycosylation is critical to driving either the anti- or proinflammatory capability of IgG (1). Glycosylation of the only available carbohydrate attachment site (Asn^297^) in the Fc is essential for interactions with type 1 receptors (Fcγ) and type 2 receptors (glycan dependent) but also for driving interactions with the complement cascade (2–5).

In humans, infusion of Fc fragments is sufficient to ameliorate idiopathic thrombocytopenic purpura in children, demonstrating the therapeutic use of the Fc in vivo (6). These anti-inflammatory properties of the Fc are lost after deglycosylation of IgG, and a population of IgG-bearing sialylated Fcs has been identified as making a significant contribution to the control of inflammation in animal models (7, 8). Higher levels of sialylation also leads to longer serum retention times (9, 10), and studies in humans and mice have shown that influx and efflux of IgG into the CNS is glycan and sialic acid dependent (11–16).

Consequently, the efficacy of sialylated Fc has generated an incentive to modify the existing glycans on Asn^297^, either by chemical means or through mutagenesis programs in the Fc protein backbone that disrupt the protein–Asn^297^–carbohydrate interface (17–19). However, chemical modification of pre-existing glycans is expensive and reliant on a sustainable source of human Fc, whereas mutagenesis approaches on the Fc, or expression in glycosidase-deficient/transgenic cell lines, have yielded little improvement in Asn^297^ sialylation to the levels required for significant enhancements in the affinity of binding to FcγRs (18, 19). Recently, coadministration of two glycosyltransferase Fc-fusion proteins has been shown to convert endogenous IgG into sialylated anti-inflammatory IgGs that attenuate autoimmune disease in animal models in a platelet-dependent manner (20). Although in vivo enzymatic sialylation may circumvent many technical issues concerned with chemical or mutagenic approaches to generating sialylated IgG, it may not be appropriate in all clinical settings, for example in neurologic diseases (e.g., neuromyelitis optica) in which the target site is mostly devoid of platelets and in which two different Fc fusions would need to traverse the blood–brain barrier simultaneously. This approach also runs the risk of off-target glycan modifications and known immunogenicity of long-term administration of Fc fusions (21).

Mutagenesis studies to date have also been limited in two further respects. Side-chain changes have typically been restricted to alanine or serine, and functionality studies have mostly been confined to FcγR-binding studies (22, 23). It is therefore of academic interest and potential clinical value to explore more thoroughly how the introduction of additional *N*-glycan sites into the Fc might affect changes in binding to FcγR and other atypical Fc glycan receptors, including sialic acid–binding Ig-type lectin (Siglecs) and C-type lectins.

We recently published two complementary approaches that radically increase the sialic acid content of the Fc (24) first by insertion of the 18-aa tailpiece from IgM onto the C terminus of the IgG1–Fc into which a cysteine-to-alanine substitution is made at Cys^575^ and second by the addition of an extra *N*-glycan to the N terminus at position Asn^221^. This approach resulted in both multimeric and monomeric molecules that are >75% sialylated (compared with 2% for the IgG–Fc control) that bind to sialic acid–dependent receptors, including Siglec-1 and myelin-associated glycoprotein (MAG) (24), which are clinically implicated in the control of neuropathology (15, 25). As many pathogens rely on glycans to infect host cells, these reagents may also be useful as inhibitors of infection (26).

**FIGURE 1.**
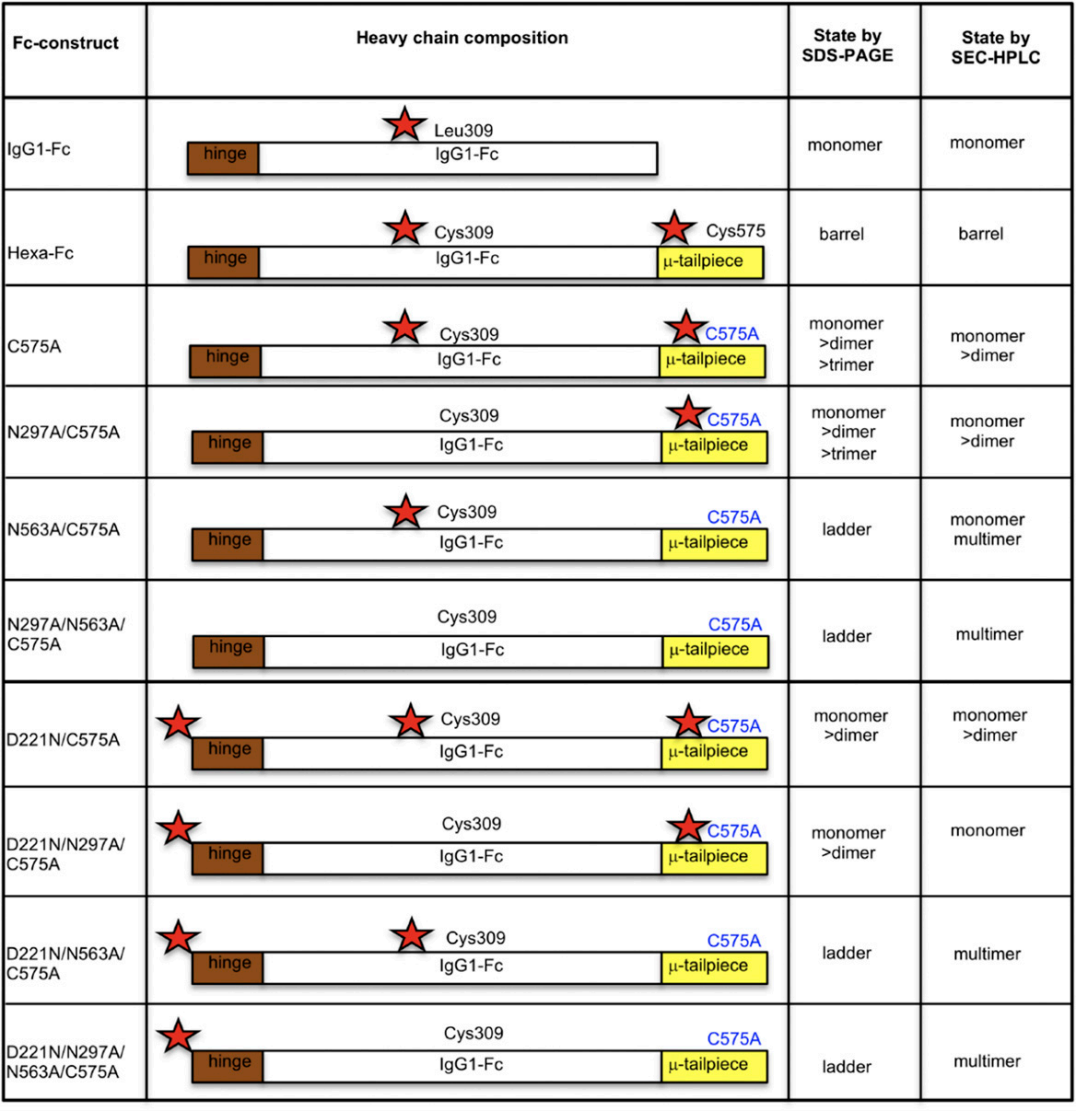
Schematic showing the various hexa-Fc glycan mutants in which Cys^575^ is mutated to alanine to create the C575A panel of mutants. Red stars indicate the hinge Asn^221^, the Cγ2 Asn^297^, and the tailpiece Asn^563^ glycan sites.

**FIGURE 2.**
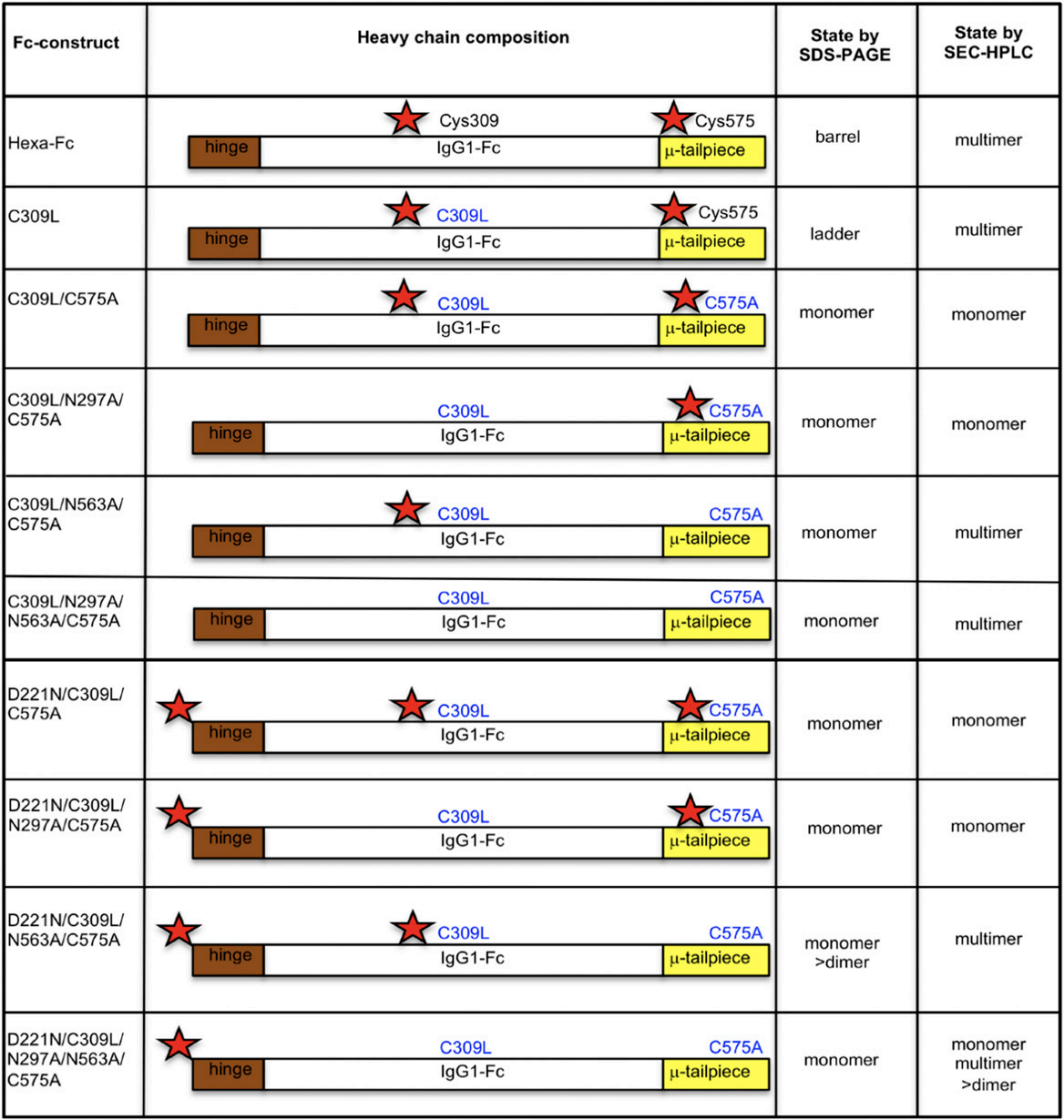
Schematic showing the C575A panel of glycan mutants from Fig. 1 in which Cys^309^ and Leucine^310^ is additionally changed to leucine and histidine as found in the native IgG1 Fc sequence to create the C309L/C575A panel of mutants. Red stars indicate the hinge Asn^221^, the Cγ2 Asn^297^, and the tailpiece Asn^563^ glycan sites.

The human IgG1–Fc typically does not bind glycan receptors because the glycan attached to Asn^297^ is largely buried within the cavity formed by the CH2-CH3 homodimer (27, 28). The location and content of glycans attached at Asn^297^ also modulates the affinity of the Fc for binding to the classical FcγRs through conformational changes imparted to the FcγR-binding region located in the lower hinge (29). In this article, we show that these limitations to Asn^297^-directed receptor binding can be overcome through a program of mutagenesis aimed at disrupting disulfide bonding while enhancing *N*-linked glycosylation within the IgG1 Fc (Figs. 1, 2).

To this end, we created two panels of human IgG1 Fc mutants (Figs. 1, 2) by deleting critical disulfide bonds and/or by inserting or deleting *N*-linked asparagine attachment sites located within the previously described IgG1–Fc multimer (2, 5, 24, 30). This approach not only yielded molecules with enhanced binding to low-affinity FcγRs but also showed interactions with receptors not previously known to bind the IgG1 Fc, including Siglec-1, Siglec-2, Siglec-3, Siglec-4, CD23, Dectin-1, Dectin-2, CLEC-4A (C-type lectin dendritic cell immunoreceptor [DCIR]), CLEC-4D, macrophage mannose receptor (MMR), mannose-binding lectin (MBL), and DEC-205. Finally, we were able to identify monomeric Fc glycan mutants with enhanced binding to influenza A virus hemagglutinin (HA) that inhibited viral-mediated agglutination of human erythrocytes.

## Materials and Methods

### Production of mutants

The generation of glycan mutants in all combinations has been described previously for the hexa-Fc that contains cysteines at both positions 309 and 575 (24). To make the new mutants described in Fig. 1 in which Cys^575^ was mutated to alanine, PCR overlap extension mutagenesis was used with a pair of internal mismatched primers 5′-ACCCTGCTTGCTCAACTCT-3′ / 3′-GGCCAGCTAGCTCAGTAGGCGGTGCCAGC-5′ for each plasmid vector coding for a designated glycan modification. The parental plasmids used for these new PCR reactions have been described previously (24). The resulting C575A mutants were then further modified to remove Cys^309^ using primer pair 5′-TCACCGTCTTGCACCAGGACT-3′ / 3′-AGTCCTGGTGCAAGACGGTGA-5′ to create the panel of double cysteine knockouts described in Fig. 2. To verify incorporation of the desired mutation and to check for PCR-induced errors, the open reading frames of the new mutants were sequenced on both strands using previously described flanking primers (24). CHO-K1 cells (European Collection of Authenticated Cell Cultures) were transfected with plasmid using FuGene (Promega), and Fc-secreting cells were cloned, expanded, and the proteins purified as previously described (2, 30).

### Receptor and complement binding assays

Methods describing the binding of mutants to tetrameric human dendritic cell–specific intercellular adhesion molecule-3–grabbing nonintegrin (DC-SIGN; Elicityl), Siglec-1, Siglec-4, and Siglec-3 (Stratech Scientific) have all been described previously (2, 30). The same ELISA protocol was used for Siglec-2, CD23, dec-1, dec-2, clec-4a, clec-4d, MBL, and MMR (Stratech Scientific or Bio-Techne). Binding of C1q and C5b-9 have been described previously (2, 30). ELISAs were used to investigate binding of Fc glycan mutants to human FcγRI, FcγRIIA, FcγRIIB, FcγRIIIA, and FcγRIIIB (Bio-Techne). Receptors were coated down on ELISA plates (Nunc) in carbonate buffer (pH 9) (Sigma-Aldrich) at 2 μg/ml overnight at 4°C, unless otherwise specified. The plates were blocked in PBS/0.1% Tween-20 containing 5% dried skimmed milk. Plates were washed three times in PBS/0.1% Tween-20 before adding Fc mutant proteins at the indicated concentrations and left at 4°C overnight. Plates were washed as above and incubated for 2 h with 1:500 dilution of an alkaline phosphatase–conjugated goat F(ab′)_2_ anti-human IgG (The Jackson Laboratory). Binding of the secondary detecting Fab’_2_ anti-human Fc was checked by direct ELISA to every mutant to ensure there were no potential biases in the detection of binding of different mutants to different receptors (Supplemental Fig. 1A). Plates were washed and developed with 100 μl/well of a SIGMAFAST *p-*nitrophenyl phosphate solution (Sigma-Aldrich). Plates were read at 405 nm, and data were plotted with GraphPad Prism.

### Binding to HA

ELISA plates were coated with 5 μg/ml recombinant HA from different influenza A and B viruses (BEI Resources) or native influenza A New Caledonia H1N1 virus (2B Scientific) in carbonate buffer (pH 9) and left at 4°C overnight. Plates were washed five times with TSM buffer (20 mM Tris-HCl, 150 mM NaCl, 2 mM CaCl_2_, 2 mM MgCl_2_) prior to blocking for 2 h in 150 μl/well of TSM buffer containing 5% BSA. After washing as before, 100 μl of Fc fragments at 30 μg/ml in TSM buffer was added in triplicate wells. Fc fragments were allowed to bind overnight at 4°C. Plates were washed five times with excess TSM buffer prior to the addition of 100 μl/well of alkaline phosphatase–conjugated F(ab′)_2_ goat anti-human IgG1 Fcγ fragment-specific detection Ab diluted 1 in 500 in TSM buffer. Glycosylated Fc fragments that bound to the glycan receptors were left to bind the conjugated Ab for 1 h at room temperature on a rocking platform. Plates were washed as above and developed for 10 min with 100 μl/well of p-Nitrophenyl phosphate. Plates were read at 405 nm using a LT-4500 Microplate Absorbance Reader (Labtech), and the data were plotted with GraphPad Prism.

### Hemagglutination inhibition assay

To determine the optimal virus-to-erythrocyte ratio, 2-fold virus stock (2B Scientific) dilutions were prepared in U-shaped 96-well plates (Thermo Fisher Scientific). The same volume of a 1% human O^+^ RBC suspension (Innovative Research) was added to each well and incubated at room temperature for 60 min until erythrocyte pellets had formed in the negative control. After quantifying the optimal virus-to-erythrocyte concentration (4HA units), serial 2-fold dilutions of Fc, control IVIG (GAMMAGARD, Baxter Healthcare), or polyclonal goat anti-influenza H1N1 (Bio-Rad Laboratories) were prepared, starting at a concentration of 2 μM, and mixed with 50 μl of the optimal virus dilution. After a 30 min incubation at 4°C, 50 μl of the human erythrocyte suspension was added to all wells and plates incubated at room temperature for 1 h, after which erythrocyte pellets could be observed in the positive controls.

### *N*-glycomic analysis

*N*-glycomic analysis was based on previously developed protocol with some modifications (31). Briefly, the *N*-glycans from 50 μg of each sample were released by incubation with New England BioLabs Rapid PNGase F and isolated from peptides using Sep-Pak C18 cartridges (Waters). The released *N*-glycans were permethylated, prior to MALDI mass spectrometry analysis. Data were acquired using a 4800 MALDI-TOF/TOF mass spectrometer (Applied Biosystems) in the positive ion mode. The data were analyzed using Data Explorer (Applied Biosystems) and GlycoWorkbench (32). The proposed assignments for the selected peaks were based on composition together with knowledge of the biosynthetic pathways.

### Binding to FcγRs by Biacore

Binding to FcγRs was carried out using a Biacore T200 biosensor (GE Healthcare). Recombinantly expressed FcγRs (R&D systems and Sino Biological) were captured via their histidine tags onto CM5 chips precoupled with ∼9000 reflective units anti-His Ab (GE Healthcare) using standard amine chemistry. Fc mutants were injected over captured receptors at a flow rate of 20 μl/min, and association and dissociation were monitored over indicated time scales before regeneration with two injections of glycine (pH 1.5) and recalibration of the sensor surface with running buffer (10 mM HEPES, 150 mM NaCl [pH 7]). Assays were visualized with Biacore T200 evaluation software v 2.0.1.

## Results

### Disulfide bonding and glycosylation influence the multimerization states of hexa-Fc

**FIGURE 3.**
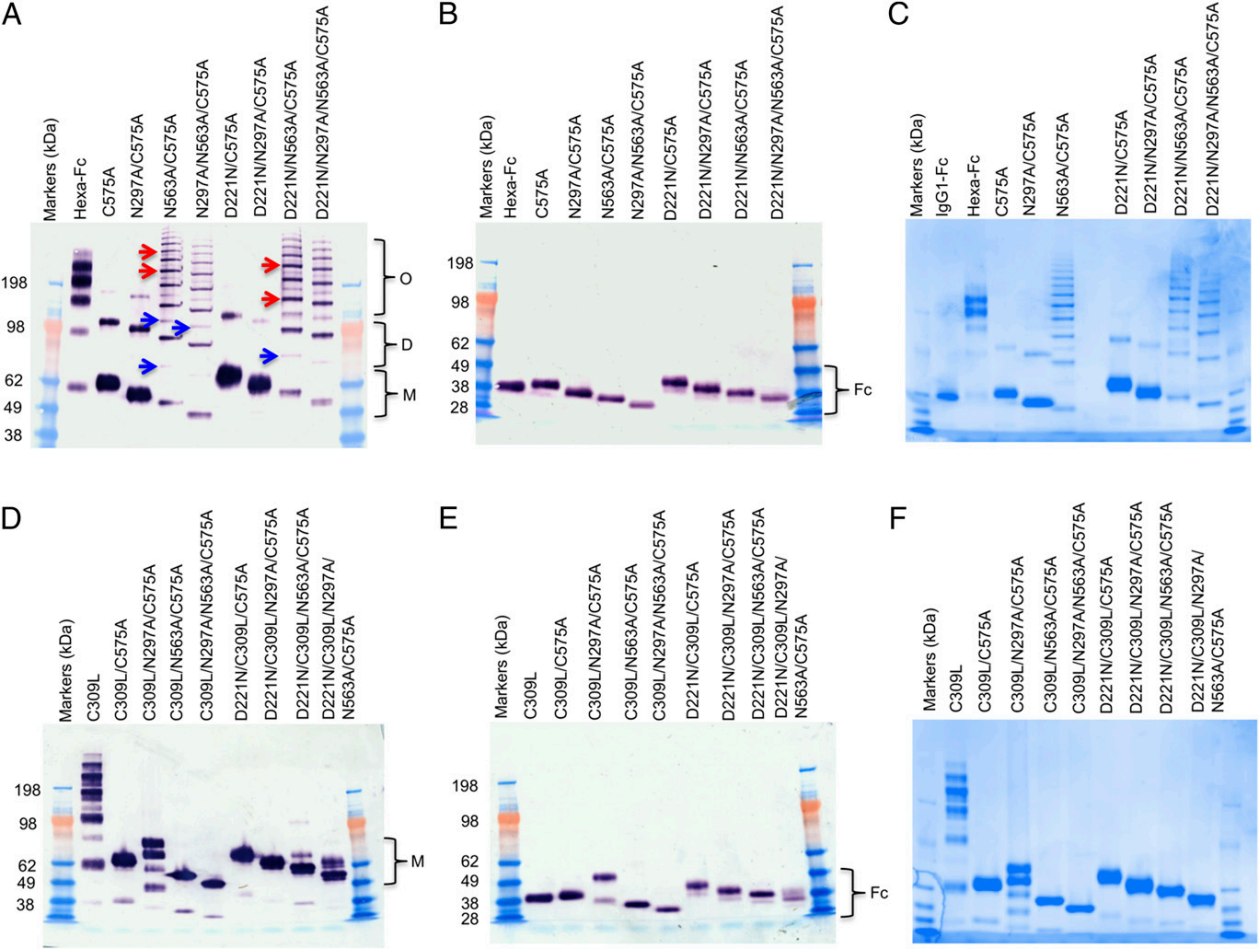
Characterization of mutant Fc proteins by SDS-PAGE. (**A**) N563A/C575A and N297A/N563A/C575A form laddered multimers (red arrows) with folding intermediates (blue arrows) that are different to those formed by the hexa-Fc control. The C575A and N297A/C575A mutants run as monomers, with dimers and trimers also seen. Removal of Asn^563^ favors multimerization in the presence of Cys^309^ but the absence of Cys^575^. The addition of a N-X-(T/S) glycan sequon to generate N-terminally glycosylated hinges (the D221N series of mutants) did not affect multimerization but increased the molecular mass of all mutants. (**B**) The same mutants as in (A) but run under reducing conditions. (**C**) The same mutants as in (A) but stained with Coomassie reagent. The decreasing molecular masses seen in the Fc represent sequential loss of *N*-linked glycans. The N297A/N563A/C575A mutant has the smallest molecular mass because it has no glycans attached to the Fc, and D221N/C575A has the largest molecular mass because it has three glycans attached. The types of glycans attached at Asn^221^, Asn^297^, and Asn^563^ for all mutants are shown in Fig. 9 and Supplemental Figs. 2–4. (**D**) Substitution of Cys^309^ with leucine onto the mutants shown in (A) to create the double cysteine knockouts, which run as monomers. Differing molecular masses are seen with C309L/N297A/C575A monomers, which may represent differential glycosylation of Asn^563^. (**E**) The same mutants as in (D) but run under reducing conditions. (**F**) Coomassie-stained gel of (D). All proteins were run under either nonreducing or reducing conditions at 2 μg protein per lane on a 4–8% acrylamide gradient gel, transferred to nitrocellulose, and blotted with anti-human IgG Fc (Sigma-Aldrich).

To determine the contribution of two *N*-linked glycosylation sites (Asn^297^ and Asn^563^) and two cysteine residues (Cys^309^ and Cys^575^) in the multimerization of hexa-Fc (2), we created two panels of glycosylation- and cysteine-deficient mutants by site-directed mutagenesis, using the previously described hexa-Fc as the template (Figs. 1, 2) (2, 24). We also inserted an *N*-linked attachment site at the N terminus of the Fc (D221N) to investigate the impact of additional glycosylation on Fc function (Figs. 1, 2). Following transfection of these mutated IgG1–Fc DNAs into CHO-K1 cells, stable clonal cell lines were established, and the secreted Fcs were purified by protein G affinity chromatography. The purified proteins were analyzed by SDS-PAGE (Fig. 3) and size-exclusion chromatography (SEC)-HPLC (Supplemental Fig. 1C).

When analyzed under nonreducing conditions (Fig. 3A, 3C and Supplemental Fig. 1C), the C575A mutant migrated mostly as monomers (∼55 kDa), with a very small proportion of dimer (∼110 kDa) and trimer (∼165 kDa). Insertion of a glycan at Asn^221^ into the C575A mutant (to create D221N/C575A) resulted in reduction of the trimer fraction and a decrease in the proportion of dimers observed, although the molecular weights of each of the species increased as a consequence of the additional N-terminally attached Asn^221^ sugar (Fig. 3A–C, Supplemental Fig. 1C).

Because we had previously shown that removal of the tailpiece glycan (Asn^563^) in hexa-Fc led to the formation of dodecamers (24), we reasoned that a similar mutation introduced into the C575A mutants would also lead to enhanced dodecamer formation. Surprisingly, removal of Asn^563^, as in N563A/C575A, N297A/N563A/C575A, D221N/N563A/C575A, and D221N/N297A/N563A/C575A, led to the formation of a laddering pattern of different molecular masses from ∼50 to >500 kDa (Fig. 3A, red arrows, 3C), representing monomers, dimers, trimers, tetramers, pentamers, hexamers, etc. Weaker bands between these species may represent 25 kDa folding intermediates that include Fc halfmers (Fig. 3A, blue arrows). All proteins in which the tailpiece Asn^563^ glycan was substituted for alanine run as multimers in solution when examined by SEC-HPLC (Supplemental Fig. 1C).

By running these mutants under reducing conditions, we were able to determine the relative sizes and occupancy of the glycans attached at each position, showing that the Asn^221^ and Asn^563^ glycans are larger than that at Asn^297^ and that fully aglycosylated null mutants such as N297A/N563A/C575A are ∼10 kDa lighter than either hexa-Fc or C575A glycan-competent molecules (Fig. 3B).

As Cys^309^ is present in these mutants (Figs. 1, 3A–C), the ladders may arise through disulfide bond formation between the only freely available sulfhydryl at Cys^309^ in two adjacent monomers. We reasoned that the loss of the tailpiece glycan in these four N563A mutants allows the hydrophobic amino acid residues (Val^564^, Leu^566^ and Ile^567^) also located in the tailpiece to cluster, thereby permitting disulfide bonding at Cys^309^.

To test the hypothesis that Cys^309^ was indeed responsible for the laddering seen with the N563A-deficient mutants, we generated a second panel of C575A mutants in which Cys^309^/Leu^310^ are mutated to Leu^309^/His^310^ as found in the wild-type IgG1 Fc sequence (Fig. 2). We also generated the mutant CL309-310LH (C309L) in which the tailpiece Cys^575^ was still present. This mutant ran similarly to hexa-Fc under nonreducing conditions, albeit with the presence of intermediates (Fig. 3D, blue arrows) that were notably absent in hexa-Fc, showing that Cys^309^ stabilizes the quaternary structure in the presence of Cys^575^.

Importantly, the loss of Cys^309^ also resulted in the loss of the ladders previously seen in the Cys^309^-competent mutants (Fig. 3D, 3F versus 3A, 3C), with all the double cysteine mutants now running principally as monomers by SDS-PAGE. The C309L/N297A/C575A mutant runs as four different monomeric species (Fig. 3D) that resolve as two bands under reduction (Fig. 3E). These bands may represent glycan variants arising at Asn^563^. Given that these variants are absent in the C309L/C575A mutant, we conclude that the presence of Asn^297^ glycan also controls glycosylation efficiency at Asn^563^. To a degree, the presence of the Asn^221^ glycan also limits the occurrence of these Asn^563^ glycoforms because under reduction, only a single band is seen in the D221N/C309L/N297A/C575A mutant (Fig. 3E).

Although the panel of double cysteine knockouts run mostly as monomers on SDS-PAGE (Fig. 3D, 3F), the double cysteine knockouts containing the N563A substitution run as a mixture of monomers and multimers in solution (Supplemental Fig. 1C). Thus, removal of the bulky Asn^563^ glycan exposes hydrophobic amino acid residues in the tailpiece that facilitate noncovalent interactions in solution that would not otherwise readily occur in the presence of the sugar.

### The Asn^297^ and Asn^563^ glycans are critical for the interactions of mutants with glycan receptors, and their absence can be compensated by the presence of Asn^221^

**FIGURE 4.**
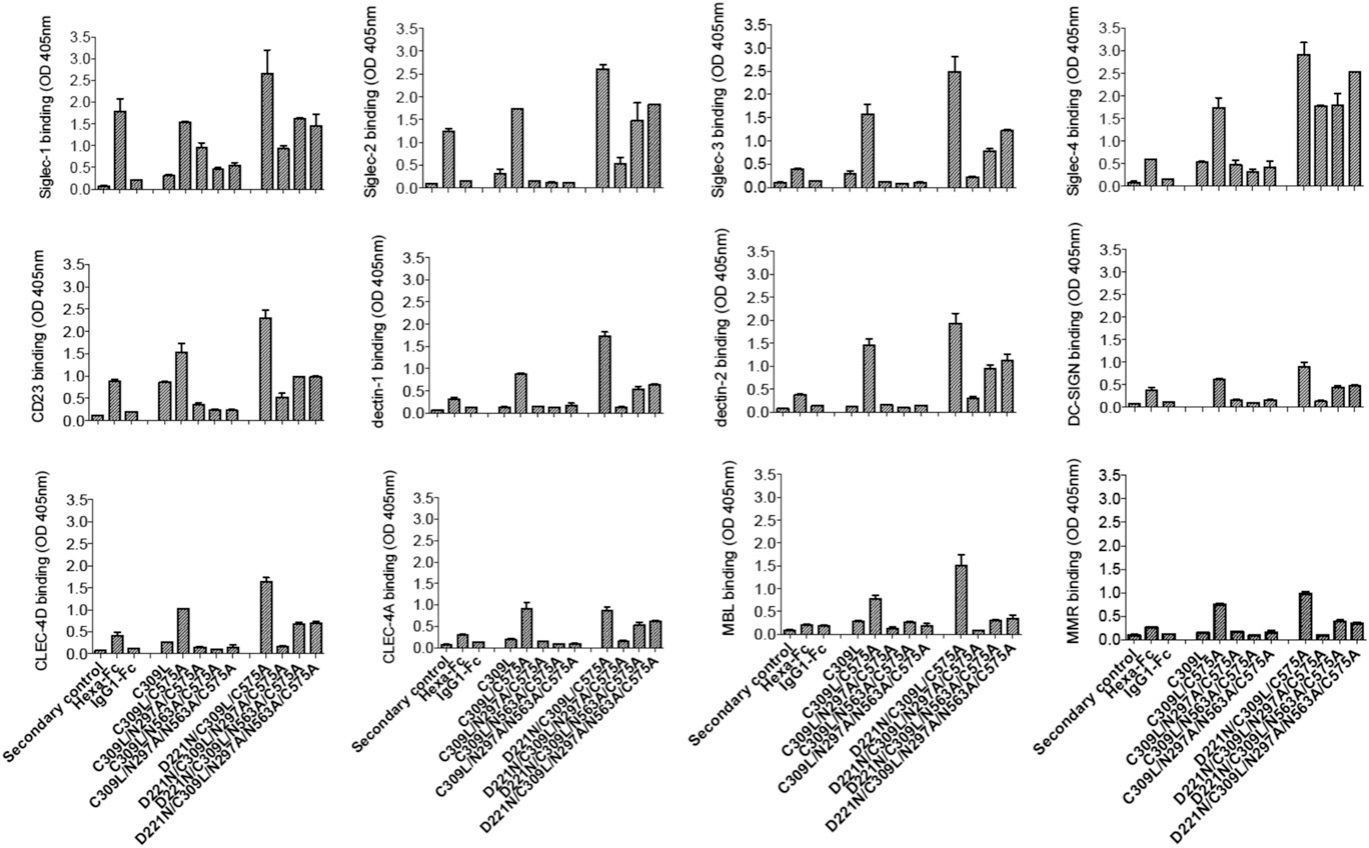
Binding of the C309L and C575A double cysteine IgG1–Fc glycosylation mutants to glycan receptors. Mutants lacking either the N297 and/or N563 glycans are severely restricted in their capacity to bind glycan receptors as determined by ELISA. The addition of an *N*-linked sugar at position 221 into the Asn^297^ and/or Asn^563^ mutants reinstates binding to all receptors investigated, with the exception of MBL, MMR, and DC-SIGN. Insertion of Asn^221^ into C309L/C575A enhances interactions to all the glycan receptors investigated. Error bars represent SD around the mean value; *n* = 2 independent experiments.

To determine which *N*-linked glycan in the double cysteine knockout mutants (Fig. 2) contributes to receptor binding, we investigated their interaction with soluble recombinant glycan receptors by ELISA (Fig. 4, Table I). In stark contrast to the IgG1–Fc control, mutants in which both Asn^297^ and Asn^563^ are present (e.g., C309L/C575A) bound all 12 glycan receptors investigated (Fig. 4). Removal of the tailpiece glycan Asn^563^, as in C309L/N563A/C575A or C309L/N297A/N563A/C575A, abolished binding to these same receptors, showing that Asn^563^ is required for glycan receptor binding.

**Table I.** Summary of mutants and their interactions with glycan receptors

Glycan Receptors	Complex Sialylated Glycans Detected	Siglec-1	Siglec-2	Siglec-3	Siglec-4	CD23	Dectin-1	Dectin-2	DC-SIGN	Clec-4A	Clec-4D	MBL	MMR	DEC-205
C309L/N297A/N563A/C575A	−	−	−	−	−	−	−	−	−	−	−	−	−	−
IgG1-Fc	−	−	−	−	−	−	−	−	−	−	−	−	−	ND
N297A/C575A	+	+	−	−	−	−/+	−	−	−	−	−	−	−	ND
D221N/N297A/C575A	+++	++	−	−	−	−/+	−	−	−	−	−	−	−	ND
C309L/N297A/C575A	+++	++	−	−	−/+	−	−	−	−	−	−	−	−	ND
D221N/C309L/N297A/C575A	+++	++	−/+	−	+++	−/+	−	−	−	−	−	−	−	ND
C575A	−/+	+	−	−	−	−/+	−	−	−	−	−	−	−	ND
D221N/C575A	+++	++	+	−	+	−/+	−	−	−	−	−	−	−	ND
C309L/C575A	−/+	+++	+++	+++	+++	+++	++	++	+	+	+	+	+	+++
D221N/C309L/C575A	+	++++	++++	++++	++++	++++	+++	+++	+	++	+	+++	+++	+++
C309L/N563A/C575A	−	−	−	−	−/+	−	−	−	−	−	−	−	−	ND
D221N/C309L/N297A/N563A/C575A	++	++	+++	++	+++	+	+	++	−/+	+	−/+	−	−	++++
D221N/C309L/N563A/C575A	+	++	+++	+	+++	+	+	+	−/+	+	−/+	−	−	ND
D221N/N297A/N563A/C575A	+++	++	−/+	+	+	+	−	−/+	−	−	+	−	−	ND
Hexa-Fc	+	++	++	−	−/+	+	−	−	+	−	−	−	−	+
D221N/N563A/C575A	+	+	−	−	−/+	−	−	−/+	−	−	−	−	−	ND
C309L	++	−	−	−	−	+	−	−	−	−	−	−	−	−
N297A/N563A/C575A	−	+	+	−	+	−	−/+	−/+	+	−	−	+	++	ND
N563A/C575A	−/+	++	−/+	−	−	−	−/+	−/+	−/+	−/+	−	−	−	ND

++++, very strong binding in all experiments; +++, strong binding in all experiments; ++, moderate binding in all experiments; +, binding in all experiments; −/+, binding in some experiments; −, no binding.

Removal of the glycan at Asn^297^, as in C309L/N297A/C575A, also abolished binding to all glycan receptors with the exception of Siglec-1. Taken together, the data show that both Asn^563^ and Asn^297^ are required for the broad glycan receptor binding seen with the C309L/C575A mutant (Fig. 4 and Table I).

With the exception of MBL, MMR, and DC-SIGN, binding by the double aglycosylated knockout C309L/N297A/N563A/C575A could be reinstated by the addition of sialylated glycans at Asn^221^, creating the mutant D221N/C309L/N297A/N563A/C575A. The Asn^221^ glycan contributes all the sialylated sugars that are required to explain the marked improvements in binding to other glycan receptors, compared with all equivalent mutants lacking Asn^221^ (Supplemental Figs. 2–4). This is in agreement with our previous work in which we demonstrated in fully cysteine-competent multimers that Asn^221^ is >75% terminally sialylated (24).

The C309L mutant that can form cysteine-linked multimers because of the retention of Cys^575^ in the tailpiece (Fig. 3D, 3F and Supplemental Fig. 1C) was unable to bind to any glycan receptors with the exception of CD23 (Fig. 4). Thus, the Asn^563^ glycans are only available for binding when attached to lower valency molecules and are buried within multimers that form either through Cys^309^-driven covalent bridging or by noncovalent clustering through multiple hydrophobic amino acids located in the tailpiece (e.g., C309L/N563A/C575A).

**FIGURE 5.**
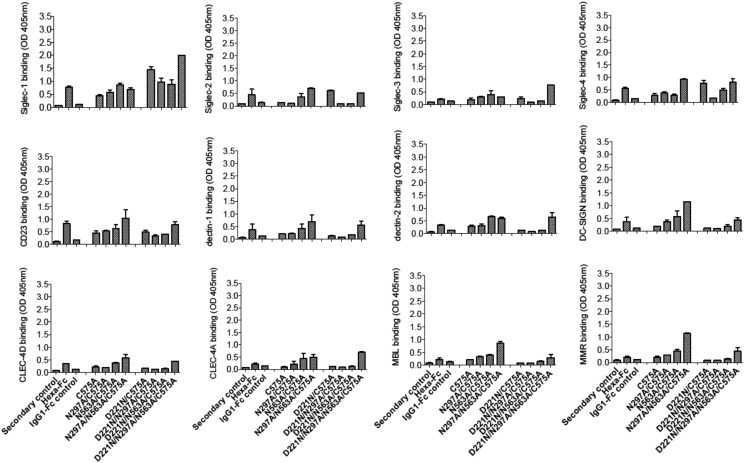
Binding of C575A mutants to glycan receptors. Proteins with a predisposition to multimerize via Cys^309^ interactions (as shown in Fig. 3A, 3C, and Supplemental Fig. 1C) are less able to engage glycan receptors than their equivalent mutants in which Cys^309^ was changed to leucine (Fig. 3D, 3F). With the exception of Siglec-1, the insertion of Asn^221^ into mutants that tend to form multimers had no effect on, or was detrimental to, binding of glycan receptors. Error bars represent SD around the mean value; *n* = 2 independent experiments.

We next investigated binding of the panel of C575A mutants in which Cys^309^ is still present (Fig. 1) and that we had shown to have the tendency to form dimers and laddered multimers (Fig. 3A, 3C and Supplemental Fig. 1C). This panel of molecules, in which disulfide bonding mediated by Cys^309^ could still occur, bound less well to all the glycan receptors investigated (Fig. 5). With the sole exception of Siglec-1, the presence of the Asn^221^ glycan was unable to improve binding, in contrast to the double cysteine knockouts. We conclude that *N*-glycans at all three attachment sites (Asn^221^, Asn^297^, and Asn^563^) are more predisposed to binding to glycan receptors when expressed on monomers and that the presence of Asn^221^ as the only glycan is sufficient to impart this broad specificity of binding, as exemplified by D221N/N297A/N563A/C575A and D221N/C309L/N297A/N563A/C575A (Figs. 4, 5).

We observed that the aglycosylated mutant N297A/N563A/C575A had a propensity to bind glycan receptors (Fig. 5). We do not have a simple answer for this observation, although the lack of binding by its counterpart C309L/N297A/N563A/C575A in which Cys^309^ is absent suggests that it may be glycan independent and a consequence of increased avidity interactions through multimerization (compare Fig. 3A v 3D).

### Glycan receptor binding is critically dependent on the presence of *N*-linked glycans

To be certain that glycan receptor binding was dependent on the presence of *N*-linked carbohydrates, and more specifically sialic acid, these sugars were removed from the triglycan D221N/C309L/C575A mutant using either PNGase F or neuraminidase (Supplemental Fig. 1B). As expected, the D221N/C309L/C575A mutant treated with PNGase F was unable to bind any of the receptors investigated, whereas treatment with neuraminidase inhibited binding to the sialic acid–dependent receptors (Supplemental Fig. 1B).

### Asn^221^-based monomers show differential binding to low-affinity human FcγRs

**FIGURE 6.**
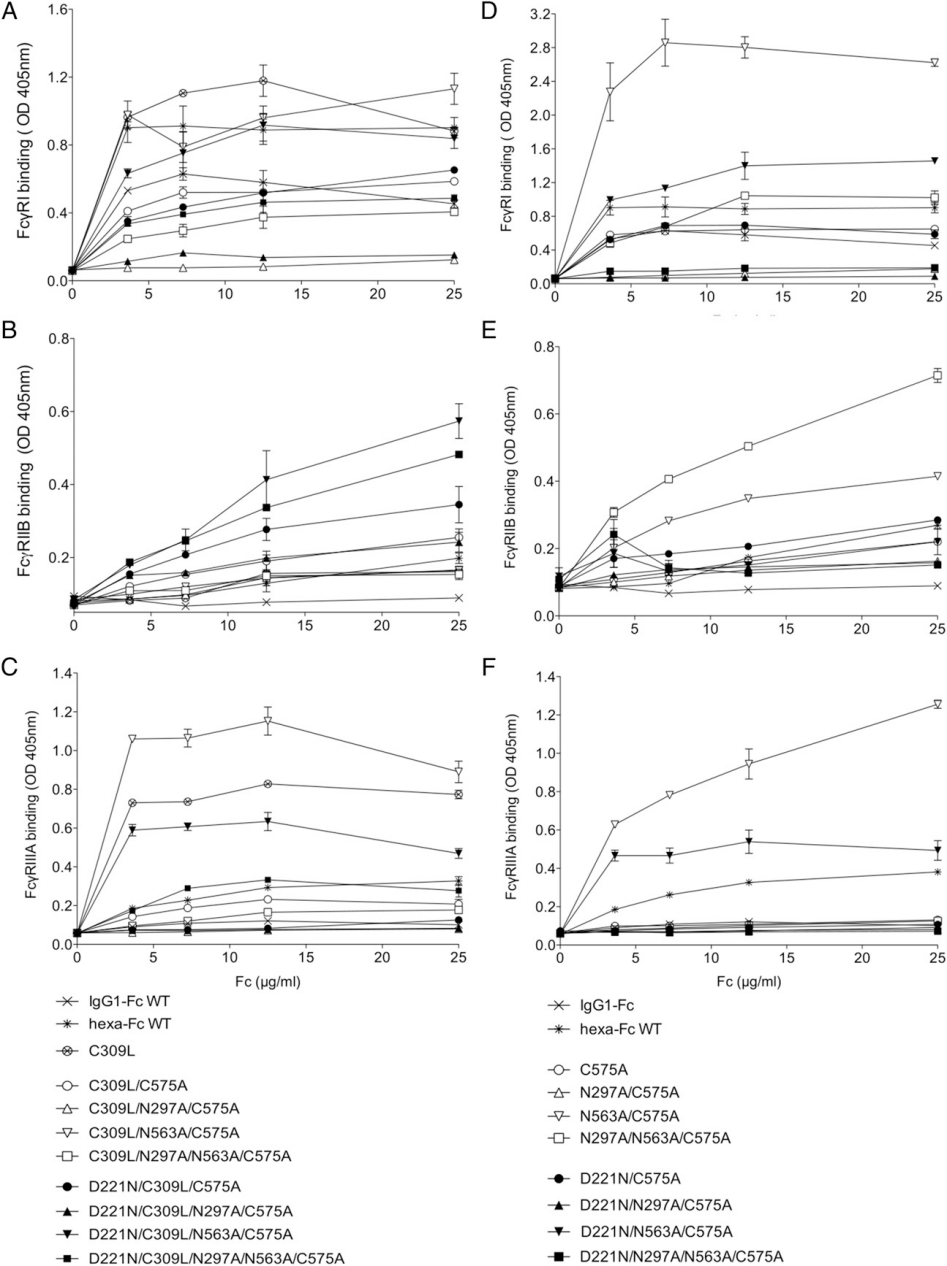
Binding of C309L (**A**–**C**) and the C575A (**D**–**F**) glycosylation mutants to classical FcγRs. The D221N/C309L/N563A/C575A mutant shows enhanced binding to FcγRI, FcγRIIB, and FcγRIIIA, whereas C309L/N563A/C575A only shows enhanced binding to FcγRI and FcγRIIIA. Mutant N563A/C575A with a predisposition to multimerize via Cys^309^ interactions (as shown in Fig. 3A, 3C) binds strongly to FcγRI and FcγRIIIA as is also seen with C309L/N563A/C575A that carries the same N563A mutation. The D221N/N563A/C575A mutant shows enhanced binding to FcγRI and FcγRIIIA. In multimers, the presence of Asn^221^ constrains interactions with FcγRIIB that are enhanced when Asn^221^ is attached to monomers (E). No improvement in binding was observed to FcγRIIA or FcγRIIIB for any of the mutants tested (data not shown). Error bars represent SD around the mean value; *n* = 2 independent experiments.

**FIGURE 7.**
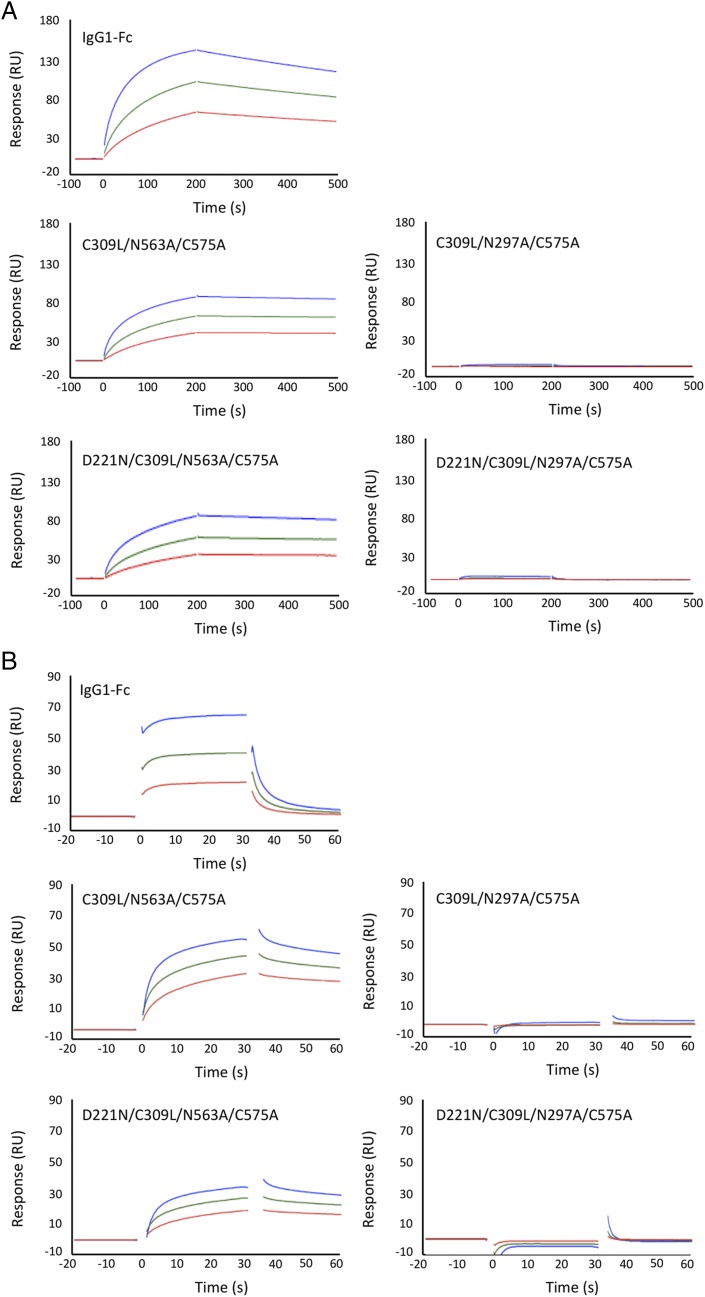
Binding of selected mutants to FcγRs by Biacore surface plasmon resonance analysis. (**A**) Binding of C309L/N297A/C575A, D221N/C309L/N297A/C575A, C309L/N563A/C575A, D221N/C309L/N563A/C575A, and monomeric Fc control to human FcγRI (CD64). Curves shown for molecules at 300, 150, and 75 nM, respectively. (**B**) Binding of the same mutants to FcγRIIIA-Val^176^ (CD32A). Curves shown for molecules at 8000, 4000, and 2000 nM, respectively. Because of the varying stoichiometry of the molecules shown (Fig. 3 and Supplemental Fig. 1C), an accurate determination of interaction kinetics is not possible. Binding to FcγRs from R&D Systems is illustrated, although binding with receptors sourced from Sino Biological gave nearly identical results.

Given the remarkable binding to glycan receptors seen with some of the glycan-modified mutants, we tested the impact that this extra glycosylation conferred on binding to the classical human FcγRs (Fig. 6, Table II). The presence of Asn^221^, for example in the D221N/C309L/N297A/N563A/C575A mutant, imparted improved binding to FcγRIIB (CD32B) even in the absence of both Asn^297^ and Asn^563^ when compared with the IgG1–Fc and controls in which Asn^221^ was absent (Fig. 6, for FcγRIIB compare filled symbols versus unfilled symbols). However, the presence of Asn^221^ did not improve binding to FcγRIIIA (compare D221N/C309L/N563A/C575A and C309L/N563A/C575A), although binding of both mutants was considerably stronger than the IgG1–Fc monomer control (Figs. 6, 7B). We hypothesize that the enhanced binding observed with the N563A-deficient mutants is a consequence of increased tailpiece-mediated assembly by all the Asn^563^-deficient proteins (Supplemental Fig. 1C). Improved binding to FcγRI was also observed with these two mutants against the IgG1–Fc control (Figs. 6, 7A), although no improvements were seen with respect to either FcγRIIA or FcγRIIIB for any of the mutants tested.

**Table II.** Summary of mutants and their interactions with Fcγ receptors

Fcγ Receptors	Complex Sialylated Glycans Detected	FcγRI	FcγRIIA	FcγRIIB	FcγRIIIA	FcγRIIIB
C309L/N297A/N563A/C575A	−	−	−	−	−	−
IgG1-Fc	−	+	−	−	−	−
N297A/C575A	+	−	−	−	−	−
D221N/N297A/C575A	+++	−	−	−	−	−
C309L/N297A/C575A	+++	−	−	−	−	−
D221N/C309L/N297A/C575A	+++	−	−	−	−	−
C575A	−/+	+	−	−	−	−
D221N/C575A	+++	+	−	−/+	−	−
C309L/C575A	−/+	+	−	−	−	−
D221N/C309L/C575A	+	+	−	+	−	−
C309L/N563A/C575A	−	++	−	−	+++	−
D221N/C309L/N297A/N563A/C575A	++	+	−	++	−/+	−
D221N/C309L/N563A/C575A	+	++	−	+++	++	−
D221N/N297A/N563A/C575A	+++	−	−	−	−	−
Hexa-Fc	+	++	+	−/+	+	−
D221N/N563A/C575A	+	++	−	−	++	−
C309L	++	++	−	−	+++	−
N297A/N563A/C575A	−	++	−	+++	−	−
N563A/C575A	−/+	++++	−	+	++++	−

++++, very strong binding in all experiments; +++, strong binding in all experiments; ++, moderate binding in all experiments; +, binding in all experiments; −/+, binding in some experiments; −, no binding.

Both the double cysteine knockouts, C309L/N563A/C575A and D221N/C309L/N563A/C575A, that form multimers in solution and bound FcγRI and FcγRIIIA (Val^176^) strongly in ELISAs were tested for binding FcγRs receptors by surface plasmon resonance analysis (Fig. 7). Both mutants displayed slower apparent off rates compared with the control Fc monomer, consistent with avidity effects either through binding to multiple immobilized FcγRs molecules or rebinding effects (Fig. 7). The loss of Asn^297^ in the C309L/N297A/C575A and D221N/C309L/N297A/C575A mutants resulted in molecules that were unable to bind FcγRs, as previously shown by ELISA (Figs. 6, 7).

We next investigated binding of the multimers formed through Cys^309^ (Figs. 1, 3A, 3C). In multimers, the presence of Asn^221^ reduced binding to all FcγRs (Fig. 6, Table II), whereas binding to the glycan receptors, although lower than that seen with monomers, was retained (Fig. 5). Multimers in which Asn^563^ and Cys^575^ are both mutated to alanine, as in N563A/C575A, bound very strongly to FcγRI and FcγRIIIA, with improved binding to FcγRIIB when compared with either the hexa-Fc or IgG1–Fc controls (Fig. 6). The aglycosylated multimer N297A/N563A/C575A bound very well to the inhibitory FcγRIIB receptor while retaining binding to FcγRI (Fig. 6).

### Asn^221^-based monomers and multimers show reduced complement activation

**FIGURE 8.**
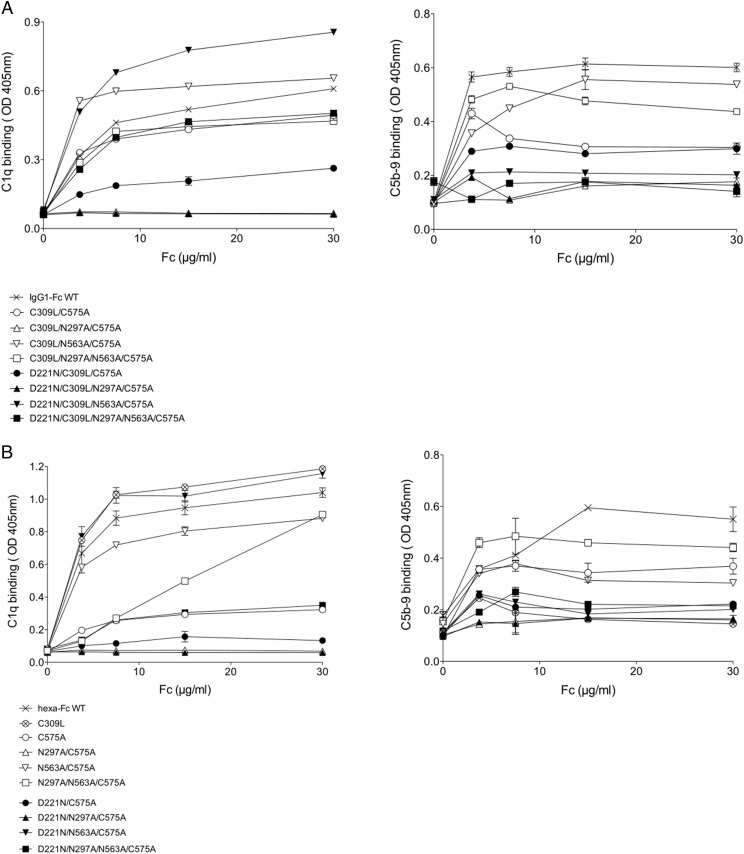
Binding of the C309L and C575A mutants to complement. (**A**) Both the C309L/N563A/C575A and C309L/N297A/N563A/C575A mutants bound C1q and permitted C5b-9 deposition. Insertion of Asn^221^ into both these mutants to create D221N/C309L/N563A/C575A and D221N/C309L/N297A/N563A/C575A allows C1q deposition but prevented subsequent C5b-9 deposition. This shows that the presence of the Asn^221^ glycan while allowing C1q to bind blocks subsequent downstream activation of the classical pathway. Mutants in which only the Asn^297^ glycan was removed, as in C309L/N297A/C575A or D221N/C309L/N297A/C575A, were unable to bind C1q or fix C5b-9. (**B**) Binding of the C575A mutants to complement. With the exception of D221N/N563A/C575A, the presence of Asn^221^ inhibited binding to C1q, although all Asn^221^-containing mutants including D221N/N563A/C575A were unable to fix C5b-9. Error bars represent SD around the mean value; *n* = 2 independent experiments.

Binding of C1q and activation of the classical complement pathway by complex monomers (Fig. 8A) and multimers (Fig. 8B) was assessed using ELISA and summarized in Table III (24, 30). With the exception of D221N/C309L/N563A/C575A, all Asn^221^-containing monomers bound C1q less well than the IgG1–Fc– or Asn^221^-deficient controls (Fig. 8A), and all four Asn^221^-containing proteins were unable to activate the classical complement pathway to its terminal components (Fig. 8A). These findings were recapitulated with the Cys^309^ mutants (Fig. 8B), including those proteins shown to form multimers (e.g., D221N/N297A/N563A/C575A against N297A/N563A/C575A). As previously shown by other groups, we have identified mutants capable of forming multimers (e.g., C309L and D221N/N563A/C575A) that avidly bound C1q but were unable to fix C5b-9 when compared with hexa-Fc (Fig. 8B) (33).

**Table III.** Summary of mutants and their interactions with complement and influenza HA

	Complex Sialylated Glycans Detected	C1q	C5b-9	Binds Native Influenza Virus (Caledonia A/H1N1)	Binds Recombinant HA (Shantou A/H3N8)	Binds Recombinant HA (Florida B)	Inhibits Influenza Virus (Caledonia A/H1N1) Agglutination
C309L/N297A/N563A/C575A	−	+	+	−	−	−	No
IgG1-Fc	−	+	+	−	−	−	No
N297A/C575A	+	−	−	−	−/+	−	ND
D221N/N297A/C575A	+++	−	−	−	++	+	ND
C309L/N297A/C575A	+++	−	−	−	+++	++	No
D221N/C309L/N297A/C575A	+++	−	−	++++	+++	+++	Yes
C575A	−/+	−/+	−/+	−	++	+	No
D221N/C575A	+++	−	−	++++	+++	++	Yes
C309L/C575A	−/+	+	−/+	−	+	−	No
D221N/C309L/C575A	+	−/+	−/+	+	+	−	ND
C309L/N563A/C575A	−	++	+	−	+	−	ND
D221N/C309L/N297A/N563A/C575A	++	+	−	+	++	+	ND
D221N/C309L/N563A/C575A	+	++	−	+	++	+	ND
D221N/N297A/N563A/C575A	+++	−/+	−	−	+	−/+	ND
Hexa-Fc	+	+++	+	+	+	−	ND
D221N/N563A/C575A	+	+++	−	−	++	+	ND
C309L	++	+++	−	−	++	−/+	ND
N297A/N563A/C575A	−	+	+	−	−	−	No
N563A/C575A	−/+	++	−/+	−	−	−	ND

++++, very strong binding in all experiments; +++, strong binding in all experiments; ++, moderate binding in all experiments; +, binding in all experiments; −/+, binding in some experiments; −, no binding.

### Asn^221^-based monomers and multimers exhibit complex sialylation patterns

**FIGURE 9.**
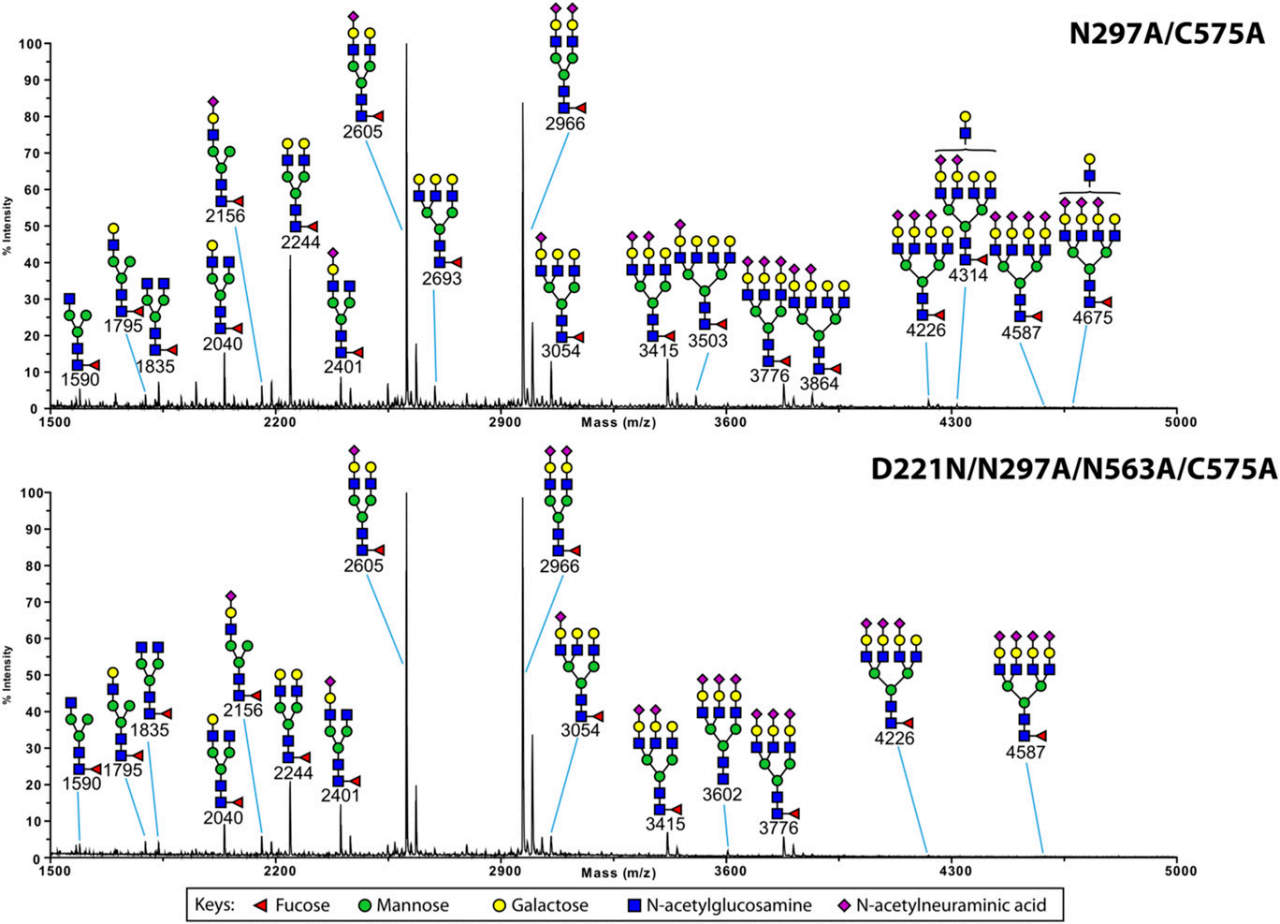
MALDI-TOF mass spectrometry profiles of permethylated *N*-glycans from N297A/C575A and D221N/N297A/N563A/C575A IgG1–Fc mutants. The data were acquired in the positive ion mode to observe [M + Na]^+^ molecular ions. All the structures are based on composition and knowledge of biosynthetic pathways. Structures shown outside a bracket have not had their antenna location unequivocally defined.

The structure of the *N*-glycan on the Fc of IgG Abs has been shown to influence multiple receptor interactions. For example, the interaction of IVIG with glycan receptors has been attributed to direct and/or indirect effects of *N*-glycan sialic acid on the Fc (29, 34, 35). Therefore, we investigated the nature of the *N*-glycans on the two panels of glycosylation- and cysteine-deficient mutants by MALDI-TOF mass spectrometry–based glycomic analysis (Fig. 9, Supplemental Fig. 2–4).

We previously demonstrated that *N*-glycans from both IgG1–Fc and clinical IVIG preparations are dominated by biantennary complex *N*-glycans with 0, 1, or 2 galactose residues (2). A minority of these complex structures are also monosialylated (2, 23, 24). Representative glycomic data are presented in Fig. 9 for N297A/C575A and D221N/N297A/N563A/C575A.

In both samples, the spectra demonstrate a higher level of *N*-glycan processing with enhanced levels of biantennary galactosylation and sialylation. In addition, larger tri- and tetra-antennary complex *N*-glycans are also observed, which can be fully sialylated (for example, peaks at m/z 3776 and 4587). Therefore, the glycomic analysis revealed that both Asn-221 and Asn-575 contained larger, more highly processed *N*-glycans that are not observed on the IgG1–Fc control (Fig. 9 and Supplemental Figs. 2–4). As predicted, no glycans could be detected on the glycosylation-deficient double mutants (N297A/N563A/C575A and C309L/N297A/N563A/C575A).

### The Asn^221^ glycan imparts enhanced binding to influenza HA

**FIGURE 10.**
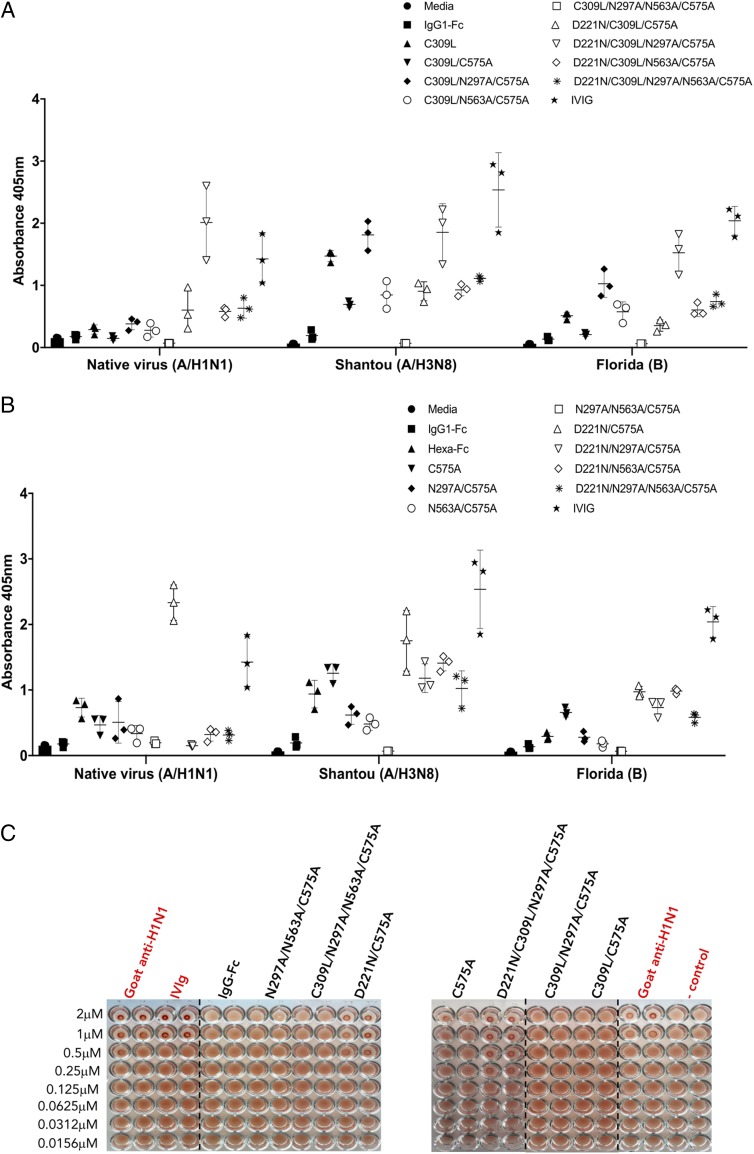
Impact of Fc glycosylation. (**A**) ELISA binding of the C309L/C575A panel and (**B**) the C575A panel of Fc glycosylation mutants to HA. (**C**) Impact of Fc glycosylation on hemagglutination inhibition. A constant amount of influenza A New Caledonia/20/99 virus H1N1 was incubated with titrated amounts of the Fc glycan mutants and added to human O^+^ erythrocytes that were then allowed to sediment at room temperature for 1 h. Nonagglutinated RBCs form a small halo. Dashed lines indicate splicing from the original plate images deposited with the journal to allow for clearer visualization of pelleted cells in each duplicated well. *n* = 2 independent experiments.

To determine if any of the hypersialylated Fc mutants possessed biologically useful properties, we investigated their binding to HA, a prototypic viral sialic acid–binding ligand (Fig. 10A, 10B). We used clinically available IVIG as a positive control because IVIG is known to contain high concentrations of IgG Abs against a diverse range of influenza HAs (36).

**FIGURE 11.**
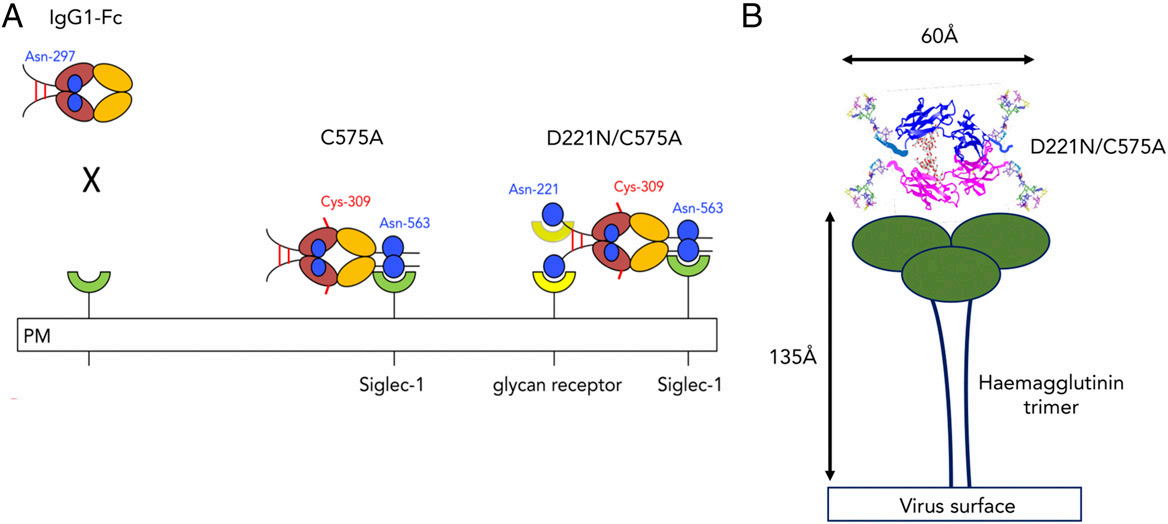
Model showing proposed *cis* interactions of the triglycan D221N/C575A mutant with (**A**) glycan receptors or (**B**) influenza HA. The glycan at Asn^297^ in the wild-type IgG1 Fc is buried and unable to interact directly with receptors. However, monomers with glycans located at both the N terminus and C terminus of the Fc (Asn^221^ and Asn^563^), as in D221N/C575A, are exposed and therefore allow crosslinking of sialic acid–dependent receptors (including Siglec-1 or HA) (48).

As expected, IVIG bound strongly to recombinant HA from both influenza A and B viruses (Fig. 10A, 10B). With the exception of the aglycosylated mutants (C309L/N297A/N563A/C575A and N297A/N563A/C575A) and the IgG1–Fc control, all the glycan-modified Fc fragments bound recombinant HA from both group A and B viruses. Binding was also reflected in the abundance of sialylated *N*-glycans of the mutant proteins (Supplemental Figs. 2–4). Thus, mutants containing Asn^221^ bound more strongly than their equivalents in which Asn^221^ was absent (Fig. 10A, 10B).

Although binding to native inactivated influenza strain A New Caledonia/20/99 virus (H1N1) was poorer than binding to either recombinant HAs from influenza A (Shantou) or influenza B (Florida), respectively, two mutants (D221N/C309L/N297A/C575A and D221N/C575A) showed superior binding to the native virus compared with either IVIG or their equivalent mutants in which Asn^221^ was absent (compare C575A with D221N/C575A) (Fig. 10A, 10B).

### Asn^221^-containing mutants inhibit hemagglutination by influenza

To test if the binding to HA has any functional relevance, we used the World Health Organization–based hemagglutination inhibition protocol to quantify influenza-specific inhibitory titers of the mutants that bound the native virus strongly (Fig. 10C). Both D221N/C309L/N297A/C575A and D221N/C575A prevented hemagglutination by New Caledonia/20/99 virus (H1N1) at concentrations as low as 0.1 μM and were demonstrably more effective than molar equivalents of either IVIG or anti-H1N1 polyclonal IgG.

In contrast, the equivalent molecules that lack Asn^221^ (i.e., C309L/N297A/C575A and C575A) failed to inhibit hemagglutination although partial inhibition was observed with the C575A mutant at the highest concentrations in some experiments (Fig. 10C). Hence, receptor binding of influenza A viruses is competed out only by mutants in which Asn^221^ and Asn^563^ are present. That both mutants run entirely as monomers by SEC-HPLC (Supplemental Fig. 1C) shows that the disposition of the glycans at the N terminus and C terminus of the Fc are more favorably orientated for binding native viral HA in monomers than multimers.

## Discussion

Many groups have postulated that multivalent Fc constructs have potential for the treatment of immune conditions involving pathogenic Abs (2, 5, 37, 38), and a recent study has shown that hexavalent Fcs can block FcγRs leading to their downmodulation and prolonged disruption of FcγR effector functions both in vitro and in vivo (39, 40). Hexameric Fcs have also been shown to inhibit platelet phagocytosis in mouse models of idiopathic thrombocytopenic purpura (33, 39, 41).

Although disulfide-bonded hexameric Fcs may provide exciting new treatment approaches to control autoimmune diseases, they are more difficult to manufacture than smaller simpler Fc molecules. Their beneficial effects must also be carefully balanced with the acute risk of proinflammatory responses observed upon FcγR crosslinking and the increased risk from infection or cancers due to long-term immune suppression. These potential drawbacks with multimeric Fcs led us to investigate if complex monomers may be developed that retain the advantages of multimers (e.g., high-avidity binding to low-affinity receptors) but that are also more readily manufactured to scale.

Although Fc engineering by mutagenesis and/or direct modification to the Asn^297^ glycan have yielded modified affinity and/or selectivity for FcγRs (1, 18, 42–47), interactions with glycan receptors have largely been ignored despite a large body of literature demonstrating their importance in controlling unwanted inflammation (48–51). However, such approaches that show enhanced receptor interactions via mutations introduced into full-length IgG molecules (3, 52, 53) may not necessarily be predictive a priori in the context of either Fc multimers or their Fc fragments (24, 39).

Furthermore, reported Fc mutations or glycan modifications have mostly focused on the conserved Asn^297^ glycan that is largely buried within the Fc (4, 17–20, 27, 28), and thus monomeric IgG1 is unable to interact with a broad range of glycan receptors (Fig. 11A). Although Siglec-2 (35), DC-SIGN (2, 54, 55), DCIR (34), and FcRL5 (2, 56) have all recently been shown to be ligands for IVIG, these interactions may also stem from specific Fab-mediated binding (57). Thus, glycosylation of intact IgG is known to be critically important, but the relative contribution of the Fc, Fab, and/or their attached glycans, together with the identity of the salient receptors involved in IVIG efficacy, remain controversial.

We took an alternative approach to glycan modification by introducing, in various combinations, two additional *N*-linked glycosylation sites (Asn^221^ and Asn^563^) into our hexa-Fc (2, 24). To investigate the effects of this additional glycosylation, hexa-Fc was further mutated to remove one (Fig. 1) or both of the cysteine residues (Cys^309^ and Cys^575^) (Fig. 2) that are required for interdisulfide bond formation between individual Fc moieties in hexa-Fc. This approach yielded complex glycosylated molecules (Figs. 3, 9 and Supplemental Figs. 2–4), including the monomeric D221N/C309L/C575A mutant that has all three glycans attached and which showed improved binding to FcγRIIB, DC-SIGN, and DCIR; these receptors being implicated in the efficacy of IVIG (Table I) (8, 17, 20, 58, 59). The triglycan mutant (D221N/C309L/C575A) also bound more strongly and broadly to all the glycan receptors investigated, including receptors recently implicated in IVIG efficacy [e.g., CD23 (60), CD22 (35), and DCIR (clec4a) (34)] when compared with monoglycosylated (e.g., IgG1-Fc) or nonglycosylated (C309L/N297A/N563A/C575A) controls (Fig. 4, Table I).

The observed binding to CD22 was particularly surprising as this receptor prefers α-2,6 linked neuraminic acid and not α-2,3 linkages attached by CHO-K1 cells, although proximity-labeling experiments have recently shown that glycan-independent interactions of CD22/Siglec-2 with Ig in the BCR is possible (61).

We also observed marked binding of D221N/C309L/C575A to dectins (Fig. 4), receptors that more typically recognize β-1,3-glucans expressed by fungal pathogens (62). Although dectin-1 is known to bind variably glycosylated human tetraspanins CD37 and CD63 (63), the anti-inflammatory activity of IgG1 immune complexes may be mediated by Fc galactosylation and associations with dectin-1 and FcγRIIB (64).

The insertion of multiple glycan sites into the Fc, in particular at Asn^221^, enables new receptor interactions that are not possible with solely Asn^297^-directed approaches (Fig. 11A). For example, we generated the di-glycan D221N/C309L/N297A/C575A mutant that displayed marked binding to Siglec-1 and Siglec-4 (MAG), both receptors being clinically implicated in the control of neuropathy (15, 25). This mutant showed no observable binding to either FcγRs or complement proteins (Tables II, III) yet was highly effective at blocking hemagglutination by influenza A virus (Fig. 10C).

As glycan-mediated binding is essential for the influenza virus to infect cells of the respiratory tract, mutations in HA that lead to loss of receptor binding are unlikely to survive any neutralizing Abs induced during an immune response (Fig. 11B). Modeling of the D221N/C575A mutant shows that the distance from the N-terminal to the C-terminal tips of the Fc is ∼60 Å (Fig. 11B), which is the same distance between the sialic acid–binding domains on the HA trimer (65). The Asn^221^ and Asn^563^ sugars located at the tips of the Fc are not constrained by their location within the Fc, as with Asn^297^, and would therefore be expected to be highly mobile and flexible with respect to searching out the HA-binding pocket.

Alternative anti-influenza therapeutic strategies are urgently needed. The use of IVIG during the 2009 and 1918 pandemics reduced mortality from influenza by 26 and 50%, respectively (66, 67), and a recent randomized, placebo-controlled study suggests these figures may be improved by enhancing influenza-specific Abs in IVIG (Flu-IVIG) preparations (36). As Flu-IVIG is manufactured in advance of future epidemics, there may be modest or no neutralizing activity against emerging strains. Combinations of Flu-IVIG or neuraminidase inhibitor drugs with Fc sialic acid–binding domain blockers may enhance the efficacy of Flu-IVIG or neuraminidase inhibitor-based medicines. Neither the D221N/C575A nor D221N/C309L/N297A/C575A mutants that inhibited hemagglutination so effectively (Fig. 10C) bind FcγRIIIA (Fig. 6 and Table II) and would thus not be expected to interfere with FcγRIIIA-dependent Ab-dependent cellular cytotoxicity toward influenza-infected cells by neutralizing IgG present in Flu-IVIG.

As well as direct HA binding, the molecules may shield sialic acid receptor binding sites on epithelial cells or act as decoy receptors through receptor mimicry, thereby preventing binding of the virus to epithelial target cells. Similarly, being rich in sialic acid, the molecules may also act as decoy substrates for neuraminidase. Intranasal delivery of Fc fragments may therefore be feasible, as Fc-fused IL-7 can provide long-lasting prophylaxis against lethal influenza virus after intranasal delivery (68). We have previously shown that Fc multimers can bind the neonatal Fc receptor (FcRn) (69). Thus, binding to the FcRn may act to increase the residence time of Fc blockers delivered to the lung (70, 71).

A potential drawback to the hypersialylation approach with respect to blocking HA may be the susceptibility of Fc glycans to viral neuraminidase. Although neuraminidase from *Clostridium perfringens* could catalyze the hydrolysis of sialic acid residues from our soluble Fc fragments and thus block interactions with glycan receptors (Supplemental Fig. 1B), it remains to be tested if HA-bound Fcs are susceptible to catalysis by the influenza neuraminidase. We believe that metabolic oligosaccharide engineering with alkyne sialic acids could create neuraminidase-resistant Fc blockers (72).

In another example, multiple mutants were shown to bind DEC-205 (Figs. 4, 5, Table I), the major endocytic receptor expressed by dendritic cells, which suggests that these constructs may be useful for the targeted delivery of Ags in vaccines. Current approaches to deliver Ag to DEC-205 rely on DEC-205–specific delivery, often with Ags fused to anti–DEC-205 mAbs (73–75), whereas approaches that target multiple dendritic cell receptors, including DEC-205, may make for more effective Ag delivery.

To be useful in vaccines, an Ag must cluster through the binding of multiple Fc regions in near-neighbor interactions with multiple low-affinity FcγRs (76), and in particular FcγRIIA, FcγRIIB, and FcγRIIIA (76–78). As described above, we generated multimers with differential binding to either FcγRIIB (e.g., N297A/N563A/C575A), FcγRIIIA (e.g., C309L and D221N/N563A/C575A), or with a capability to bind both FcγRIIB and FcγRIIIA (e.g., N563A/C575A). Multimers formed by the N563A/C575A or C309L/N563A/C575A mutants may be particularly relevant, as these were also able to bind type 2 glycan receptors and activate the complement cascade, both implicated in the efficacy of vaccines (5).

We also created molecules disrupted for covalent bonding (the double cysteine knockouts) that formed multimers in solution through noncovalent tailpiece clustering (e.g., C309L/N563A/C575A and D221N/C309L/N563A/C575A) that showed enhanced interactions with FcγRs, in particular FcγRIIIA (Figs. 6, 7). Whether these will be more effective than covalently stabilized Fcs (e.g., N563A/C575A) at enhancing FcγRIIIA-mediated effector functions, in for example therapeutic mAbs or Fc-fusion therapies/vaccines, remains to be determined.

As summarized in Tables I–III, we identified the following: 1) mutant Fc molecules that are capable of binding C1q and activating complement but that show little or no detectable interaction with either FcγRs or glycan receptors; 2) molecules with enhanced activation of complement, improved binding to FcγRs, and little engagement of glycan receptors; 3) molecules with enhanced binding to C1q but little C5b-9 deposition that retain interaction with both Fcγ and glycan receptors; and 4) monomeric molecules with enhanced binding to a subset of sialic acid–dependent glycan receptors, in particular Siglec-1, Siglec-4, and HA, with little or no interaction with either FcγRs or complement.

Consequently, by adding or removing glycosylation and/or disulfide-bonding sites within our original hexameric Fc platform (2, 5, 24), new repertoires of desirable binding attributes can be made. These molecules may be useful in the control of other pathogens, including Newcastle disease virus, group B streptococci, *Streptococcus pneumoniae*, and *Mycoplasma genitalium*, in which sialic acid–dependent interactions are also crucially important (79).

## Supplementary Material

Data Supplement
