# Supplementary material for: Insertion of N-Terminal Hinge Glycosylation Enhances Interactions of the Fc Region of Human IgG1 Monomers with Glycan-Dependent Receptors and Blocks Hemagglutination by the Influenza Virus
Source: J Immunol. 2019 Jan 25;202(5):1595–611. doi: 10.4049/jimmunol.1801337 (PMC6379808; doi:10.4049/jimmunol.1801337)
Supplement: Data Supplement [file JI_1801337.zip › JI_1801337_Supplemental_Figures_1.pdf]

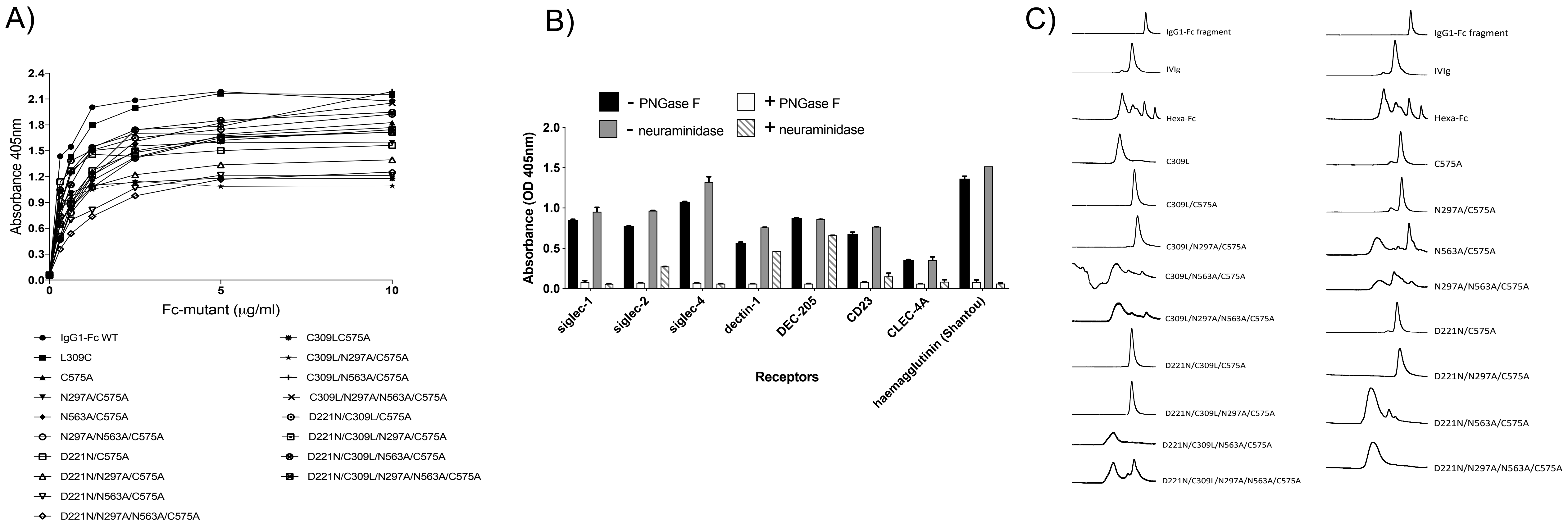

Supp. Fig 1. (**A**) The polyclonal Fab'$_2$-detecting antibody binds to all the Fc mutants used in this study. Mutants titrated down on ELISA plates and detected with a 1:500 dilution of the alkaline-phosphatase conjugated goat Fab'$_2$-anti-human Fc as described in materials and methods. (**B**) Binding to glycan receptors after cleavage with either PNGase F or neuraminidase. ELISA plates were coated with receptors at 5 μg/ml in carbonate buffer pH9 and incubated overnight at 4°C prior to blocking in TSM (20 mM Tris-HCl, 150 mM NaCl, 2 mM $CaCl_2$, 2 mM $MgCl_2$, 5% BSA) buffer pH 7.4. Wells were washed with TSM before addition of 100 μl digested (+) or undigested (-) D221N/C309L/C575A mutant at 20 μg/ml in TBS buffer to duplicate wells. After 4h incubation and washing as above, 1:500 dilution of the alkaline phosphatase-conjugated Fab'$_2$ anti-human Fc (Jackson) was added prior to washing and developing as per methods. PNGase F and α2-3,6,8 neuraminidase purchased from NEB and used as per manufacturer's instructions. (**C**) SEC-HPLC chromatograms as described in methods for the C309L/C575A and C575A panel of mutants.

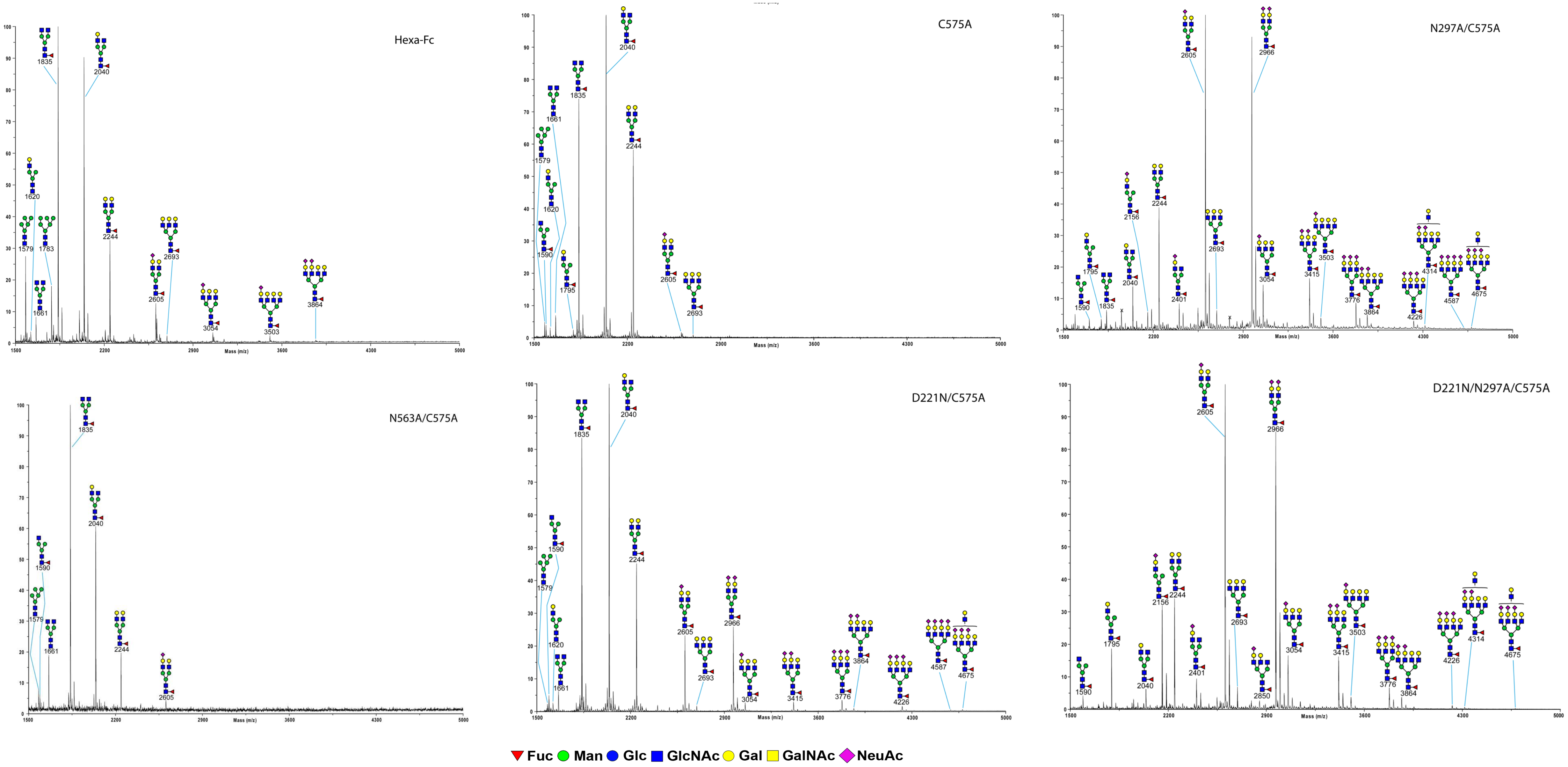

Supp. Fig 2. MALDI-TOF MS profiles of permethylated N-glycans from IgG1-Fc mutants. The data were acquired in the positive ion mode to observe [M + Na]+ molecular ions. All the structures are based on composition and knowledge of biosynthetic pathways. Structures shown outside a bracket have not had their antenna location unequivocally defined.

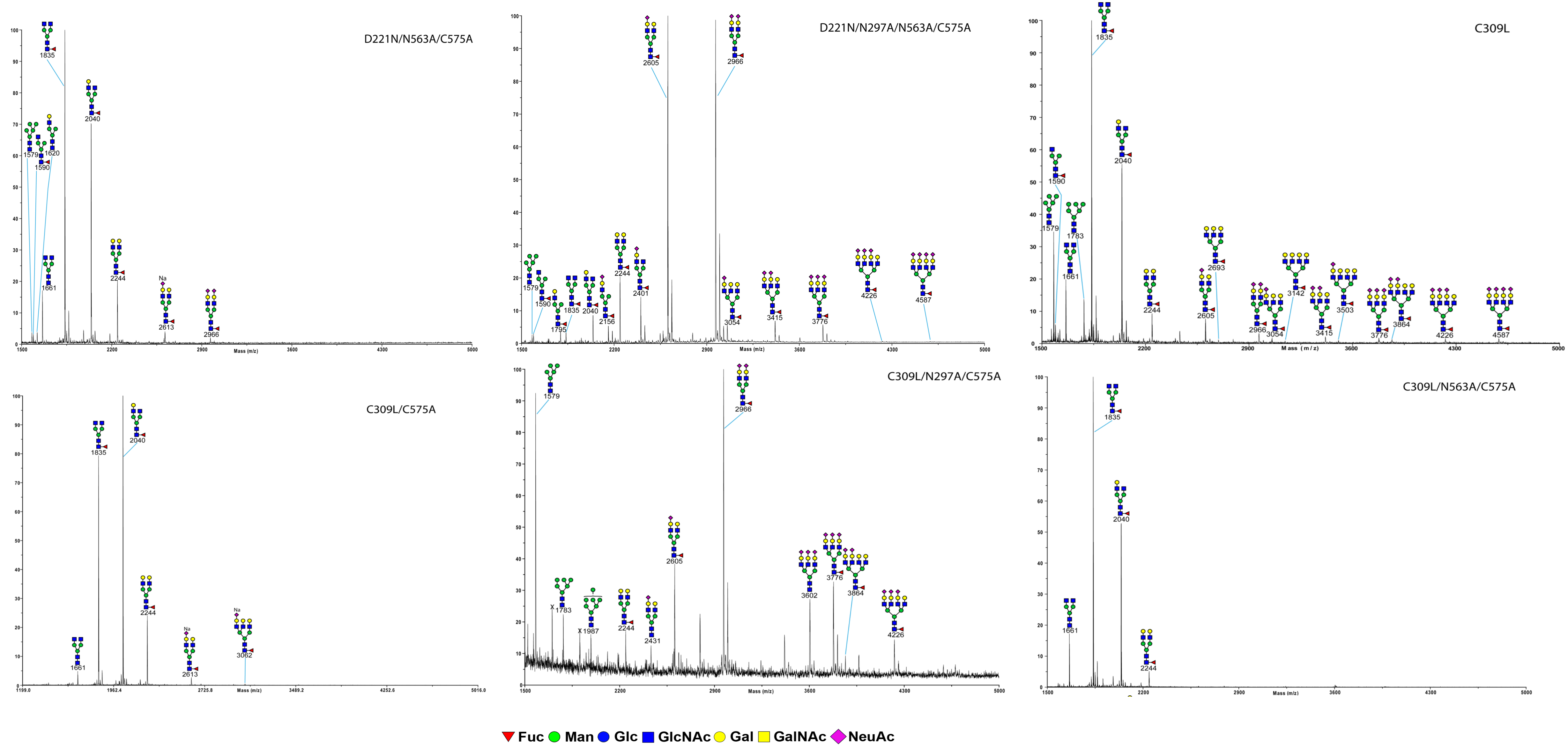

Supp. Fig 3. MALDI-TOF MS profiles of permethylated N-glycans from IgG1-Fc mutants. The data were acquired in the positive ion mode to observe [M + Na]+ molecular ions. All the structures are based on composition and knowledge of biosynthetic pathways. Structures shown outside a bracket have not had their antenna location unequivocally defined.

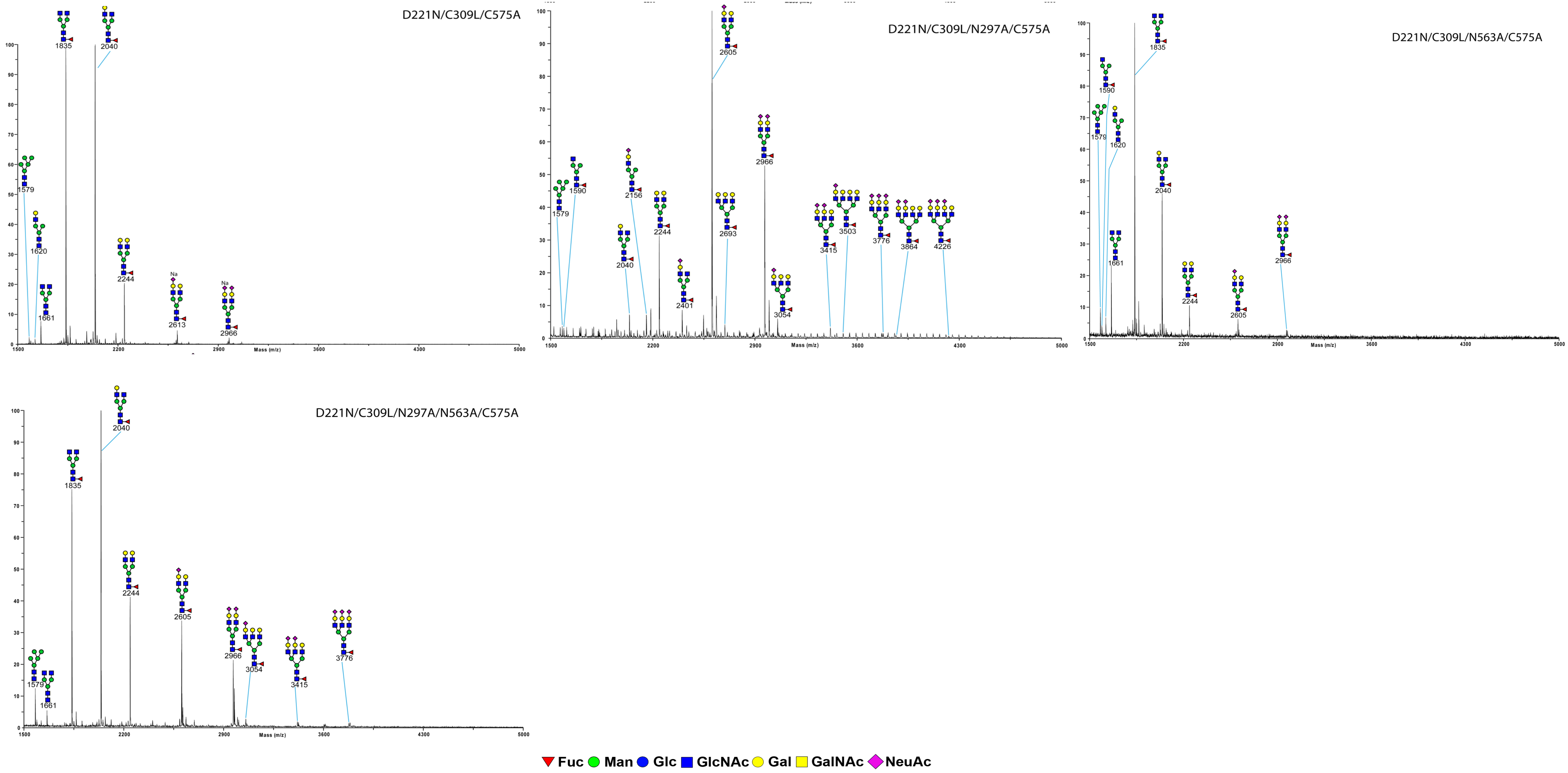

Supp. Fig 4. MALDI-TOF MS profiles of permethylated N-glycans from IgG1-Fc mutants. The data were acquired in the positive ion mode to observe [M + Na]+ molecular ions. All the structures are based on composition and knowledge of biosynthetic pathways. Structures shown outside a bracket have not had their antenna location unequivocally defined.
